# *Euglena gracilis* as a high-throughput screening platform for antibacterial activity, cytotoxicity and membrane permeability in a one-step and cost-effective assay

**DOI:** 10.1038/s41429-026-00911-5

**Published:** 2026-03-18

**Authors:** Leticia Pereira, Lea-Sophie Löffler, Susanne H. Kirsch, Marc Stadler, Birthe Sandargo, Fabiola Holetz, Rolf Müller, Susanne Kramer

**Affiliations:** 1 Biocenter, University of Würzburg, Würzburg, Germany; 2 Helmholtz Institute for Pharmaceutical Research Saarland (HIPS), Saarbrücken, Germany; 3 German Centre for Infection Research (DZIF), Partner Site Hannover-Braunschweig, Braunschweig, Germany; 4 Helmholtz Centre for Infection Research (HZI), Braunschweig, Germany; 5 Carlos Chagas Institute (ICC), FIOCRUZ/PR, Curitiba, Brazil; 6 Department of Pharmacy, Saarland University, Saarbrücken, Germany

**Keywords:** Drug screening, High-throughput screening

## Abstract

There is an urgent need for the development of new antibacterial drugs, caused by the increasing number of resistances. The first step for the development of new antibacterial compounds is usually a high-throughput screen of naturally occurring or synthetic compounds. Here, we propose to screen compound libraries on the Euglenoid *Euglena gracilis*. This protozoan has obtained a chloroplast via secondary endosymbiosis of an alga, but still maintains its ability to metabolise organic carbon sources. Importantly, the chloroplast has preserved some bacterial features and can be targeted by a range of antibiotics, resulting in organelle loss and *Euglena* bleaching, while the mixotrophic metabolism ensures growth of *Euglena*. Therefore, *Euglena* allows simultaneous screening of a compound library for (i) antibacterial activity (*Euglena* bleaches), (ii) absence of toxicity (*Euglena* grows) and (iii) membrane permeability across at least three membranes of both eukaryotic and prokaryotic origin. We have established and optimised all the parameters needed for such an *Euglena*-based antibacterial drug screen. We define absorbance parameters to distinguish the three possible outcomes (bleaching, growing and dying) in an automatised way. We have successfully tested the screen on both a commercially available compound library from MedChemExpress and on a self-assembled library of rare natural compounds from myxobacteria and fungi. Our *Euglena*-based screening platform provides a novel high-throughput method to screen for new compounds with antibiotic properties, in a cost-effective way and with any library. While some classes of antibiotics will be missed, the screen is unbiased and has the potential to discover novel antibiotic targets.

## Introduction

The discovery of antibiotics was a major medical milestone that massively decreased the death toll of infectious diseases in the 20th century, significantly contributing to the increase in life expectancy. However, it is now clear that the battle against bacterial infections is far from won, as bacteria continue to develop resistances. This process is accelerated by the excessive and/or inappropriate use of antibiotics in humans, as well as by their widespread application in industrial livestock production. Currently, 15% of all infections requiring hospitalisation are caused by multidrug-resistant pathogens and for some of these, no effective antibiotics are available [1]. Globally, infections with resistant bacteria represent the third leading cause of death, accounting for ~5 million fatalities annually [2]. The absence of effective antibiotics would not only leave us defenceless against bacterial infections once again, but would also render surgeries, caesarean sections and certain cancer treatments impossible.

In comparison to other fields of drug discovery, the discovery of antibacterial drugs is lacking behind [3, 4]. In fact, between 2004 and 2009, only a single new antibiotic per year reached clinical application [5] and the numbers have only marginally improved since. After the random discovery of penicillin by Alexander Fleming in 1928 [6], the search for new antibiotics was mainly based on the WAKSMAN platform: in the 1940s, Selman Waksman discovered streptomycin by screening soil actinomycetes for their ability to kill bacteria on an agar plate [7]. This launched the golden age of antibiotics discovery, which identified most of the antibiotic classes that are in use today. However, within less than two decades, most naturally occurring antibiotic classes had been discovered and the Waksman platform produced no new hits. For a while, synthetic compounds increased the available antibacterial drug arsenal, for example, the fluoroquinolones, which target the bacterial DNA gyrase and topoisomerase IV. However, from 1965 onwards, only a few synthetic compounds made it to the market. Moreover, target-specific drug screens that had become highly successful in other areas of drug-discovery, proved largely disappointing in the field of the discovery of antibacterial drugs and optimisation [8–10] because (i) the high costs and the call to use it only as a last-resort made the development non-attractive and (ii) broad band antibiotics are still preferred by many physicians, in particular for fast-progressing diseases with slow diagnostics.

Today, scientists are aware of the urgent need for new drugs to treat bacterial infections and are looking for new ways to screen for antimicrobial compounds. A promising way is to explore new sources for naturally occurring antibiotics. In fact, all naturally occurring antibiotics originated almost exclusively from one single group of bacteria, the actinomycetes; even the fungi-derived penicillin genes were likely transmitted from actinomycetes to fungi by horizontal gene transfer [11]. Thus, the majority of bacteria remains massively undermined for antibiotics; this affects in particular the 99% of the Earth's microbiome that cannot be cultured [12]. Moreover, most antibacterial drugs target only one of three pathways: transcription, translation or cell wall synthesis [9, 13]. Again, this leaves many bacteria-specific vulnerabilities untargeted. One example for a new target is the bacterial cell membrane, which has the additional advantage of not being directly genetically encoded and resistances is thus less likely. Third, most drugs belong to one of only a few chemical classes, leaving many chemical classes unexplored.

Most searches for antibacterial activity have one thing in common: initially, a huge compound library is screened, which then feeds a much smaller number of leads into more advanced testing platforms. We here provide a novel, efficient and cost-effective approach for this first large-scale screening step in the antibacterial drug development pipeline. At the core of this method is *Euglena gracilis*, a eukaryotic unicellular organism that acquired a chloroplast through secondary endosymbiosis of a *Pyramimonas*-related green alga. Unlike the chloroplasts of plants, the *Euglena* chloroplast is therefore surrounded by three cell membranes rather than two (it should be four, but one got lost during evolution). Importantly, *E. gracilis* is a mixotroph, which means it has never become fully dependent on photosynthesis but has preserved all metabolic pathways required for a heterotrophic lifestyle. In the absence of light, *E. gracilis* metabolises carbohydrates from the growth medium and quickly loses its chloroplast and thus its green colour, yet continues to proliferate. This loss of green colour is called bleaching. In the 1940s, it was demonstrated that bleaching can also be caused by the antibiotics streptomycin [14, 15] and later on many other antibiotics were discovered to have the same effect [16–31]. This bleaching phenomenon can be explained by the evolutionary origin of chloroplasts from bacteria: despite the long evolutionary distance, the chloroplast has still preserved some bacterial features, including being a molecular target for some antibiotics. In particular, antibiotics that inhibit DNA or protein synthesis cause *Euglena* to bleach [19]. Based on this principle, our assay for the identification of novel antibacterial drugs is straightforward: we will screen unknown compounds for their ability to induce bleaching in *Euglena gracilis*, while not killing it. In short, our assay will identify compounds with potential antibacterial activity that are likely non-toxic towards eukaryotes and potentially able to cross membranes of both prokaryotic and eukaryotic origin.

## Material and methods

### Culture of Euglena gracilis

*E. gracilis* cells were grown in Cramer-Myers medium [32]. The medium was produced by mixing the stock solutions CM-1 (100x stock = 10 g (NH_4_)_2_HPO_4_, 10 g KH_2_PO_4_, 73.5 g Trinatriumcitrate x 2H_2_O; pH 4.9 with HCl; sterilised by filtration), CM-2 (100x stock = 0.26 g CaCl_2_ x 2H_2_O; autoclaved), CM-3 (100x stock = 2 g MgSO_2_ x 7H_2_O, autoclaved), CM-4 (1000x stock = 0.3 g Fe_2_(SO_4_)_3_ × 7H_2_O, 0.15 g MnCl_2_ x 2H_2_O, 0.13 g CoCl_2_ x 6H_2_0; autoclaved), CM-5 (10,000x stock = 0.4 g ZnSO_4_ x 7H_2_O; autoclaved), CM-6 (10,000x stock = 0.2 g Na_2_MoO_4_ x 2H_2_O; autoclaved) and CM-7 (100,000 x stock, 0,2 g CuSO_4_ x 3H_2_O), to give 1x in the final medium. The pH was adjusted to 6.9 and the medium was autoclaved. Before use, the medium was complemented with vitamin B1 (2000x stock = 0.2 mg/ml), vitamin B12 (= 2000x stock = 1 µg/ml) and ethanol (0.8%).

*E. gracilis* was cultured at 28 °C in an incubator with a 12 h light/dark cycle or alternatively, at room temperature close to the window, using vented cell culture flasks for suspension cells or 96-well plates. During the drug-screening experiments, when cells were cultured in small volumes of 200 µl over several days, we used a humid chamber to reduce evaporation. The corresponding author will provide and ship Euglena cells to any lab that is interested. Alternatively, *Euglena gracilis* cultures are readily available in various online shops, for example, they are sold as teaching objects for schools.

### Compound libraries

We used a commercial compound library from MedChemExpress: all details (including concentrations and solvents) can be found in Table S1. A subset of 88 structurally diverse natural products in early stages of development (preclinical phase) was selected from the compound collection of the Helmholtz Institute for Pharmaceutical Research Saarland (HIPS); details are in Table S2. The selection primarily comprised 56 compounds derived from myxobacteria, representing a rich source of rare and bioactive secondary metabolites with unique structural features and mechanisms of action. For comparative purposes, 32 additional compounds of fungal origin were included. All compounds were prepared as 10 mM stock solutions in DMSO and tested at a final concentration of 100 µM in the screening assays. To ensure broad coverage of biological activities, compounds with diverse or yet unknown modes of action were included, while ‘classical’ inhibitors of cell wall biosynthesis were excluded.

### Drug screen experiments

An *Euglena* culture in a stationary phase (last subbed between 1-4 weeks ago) was counted in a Neubauer chamber and usually has cell densities of around 4–5 × 10^6^ cells/ml. Cells were diluted to 3 × 10^4^ cells/ml in Cramer Myers medium and plated to white-clear-bottom 96-well plates (non-treated surface, Thermofisher 265302) using a multi-pipette, 190 µl per well. The compounds were added in 10 µl volumes. The following controls were included to each plate: (i) Cramer Myers medium without cells for background correction (ii) cells without any compound as positive control (iii) cells with phleomycin (a bleomycin-family glycopeptide antibiotic produced by *Streptomyces verticillus* that mainly kills by causing oxidative DNA strand breaks) as a control for killing (iv) cells with streptomycin as a control for bleaching and (v) cells with DMSO as a control for the solvent. The plates were incubated in a humid chamber in the incubator (28 °C with 12 h light/dark cycle) for 4 days. Alternatively, incubation at room temperature close to the window is possible and takes about 7 days. At the end of incubation, absorption profiles were measured using the Tecan plate reader, from 380 to 720 nm. In the future, measurements at A680 and A530 nm should be sufficient to distinguish killing, bleaching and growing. In most experiments, assays were performed in triplicate.

## Results

### Establishment of the assay

**Fig. 1:**
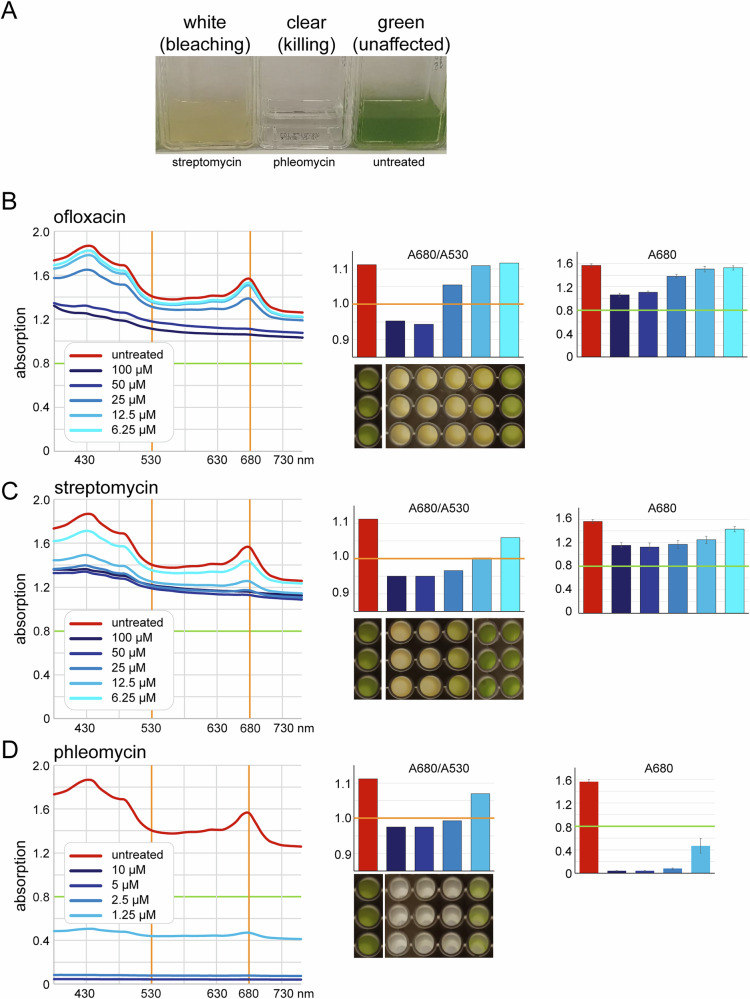
Assay establishment. **A** *Euglena* cells were seeded at 4 × 10^4^ cells/ml and treated with either streptomycin (100 µM), phleomycin (10 µM) or left untreated. The cultures are clearly distinguishable by their different colours (white, clear, green) at the end of the experiment (in this case, after seven days, culturing at room temperature). **B**–**D** *Euglena* cells were seeded at 4*10^4^ cells/ml on 96-well plates and treated with titrations of different antibiotics, in triplicate. Untreated cells served as controls. Absorption spectra were measured and are shown as averages of the triplicates (left). The bleaching factor (A680/A530) and A680 (error bars indicating standard deviations of the triplicates) are blotted for each spectrum (right)

The basic idea of the drug-screen is to seed *Euglena* cells at low cell density, add compounds from a library and let the *Euglena* grow. The three possible colours at the end of the experiment are green (‘growing’: compound has no effect), white (‘bleaching’: compound potentially has antibacterial activity) and clear (‘killing’: compound kills eukaryotes and is likely not a a good candidate for an antibacterial drug) (Fig. 1A). These outcomes need to be distinguished automatically and in high-throughput.

We first optimised all parameters. We plated *Euglena* in 96-well plates, added control antibiotics with known effects (bleaching or killing) and attempted to distinguish clear, white and green *Euglena* via absorbance profiles on a plate reader. We compared different types of 96-well plates and found that both transparent plates and white plates with optical bottom work, with the white plates giving slightly more reproducible results, as cross-talk between wells is avoided. Plates do not need to be sterile and they should not be treated for adherent cells, as this causes *Euglena* to adhere to the wells. To optimise the seeding density, we plated cells at different densities and left them either untreated or added the bleaching-antibiotic streptomycin. We found that cells need to be diluted to a cell density low enough to show no absorbance, to get a clear distinction between green and white cells at the end of the experiment (Fig. S1). An optimal cell density for seeding was 3 × 10^4^ cells/ml, which was used for all subsequent experiments. Cells were usually seeded from a dense culture in the stationary phase and it did not matter how old the culture was (within reason). Importantly, the seeding density works for different culture conditions and does not need to be adapted. *Euglena* grow best at 28 °C and a 12 h light/dark rhythm and, under these conditions, the final reads of the plate can be done after 4 days. However, if no incubator with a light source is available, the experiments can still be done, simply by reading the plates later: for our initial experiments, we left the cells growing on the windowsill at room temperature and read the plates after 7 days (e.g. Fig. S1). Moreover, we found that the exact time of reading the plates is not critical for the outcome, as long as it is long enough to get a sufficient absorbance. Thus, plate-reading can be postponed for a few days (weekend, holidays) with no change in the results. Thus, overall, we found *Euglena* screens rather robust and they can be done in all laboratories with standard molecular biology equipment.

To define the optimal wavelengths for the automatized distinction between clear, white and green cells, we treated *Euglena* cells with titrations of two bleaching antibiotics (streptomycin and ofloxacin) and one killing antibiotic (phleomycin) and measured the absorbance profiles from 380 to 760 nm (Fig. 1B, C, D). Untreated cells served as controls. The largest difference between the spectra of green and bleached *Euglena* was the disappearance of the 680 nm absorption peak in bleached *Euglena*. Instead, bleached *Euglena* would have an almost linear decline of absorbance from low to high wavelength. Therefore, we defined the bleaching factor as A680/A530: a value larger than 1 indicates green Euglena (growing) and a value smaller than 1 indicates white *Euglena* (bleaching). Killing is distinguished from bleaching by a much smaller overall absorption. With our experimental conditions, we arbitrarily defined killing by an A680 value of less than 0.8, which corresponds to about half of the A680 absorption of green *Euglena*. With different experimental conditions, the A680 of untreated *Euglena* may differ and the threshold needs to be adapted accordingly.

To summarise, cells with an A680 value of less than 0.8 will be called ‘clear’ (indicating the compound kills). If the A680 value is larger than 0.8, cells will be called ‘green’ if A680/A530 is > 1 (indicating a non-effective compound) and ‘bleached’ if A680/A530 is ≤1 (indicating an antibacterial drug candidate).

### Screen on a MedChemExpress compound library

**Fig. 2:**
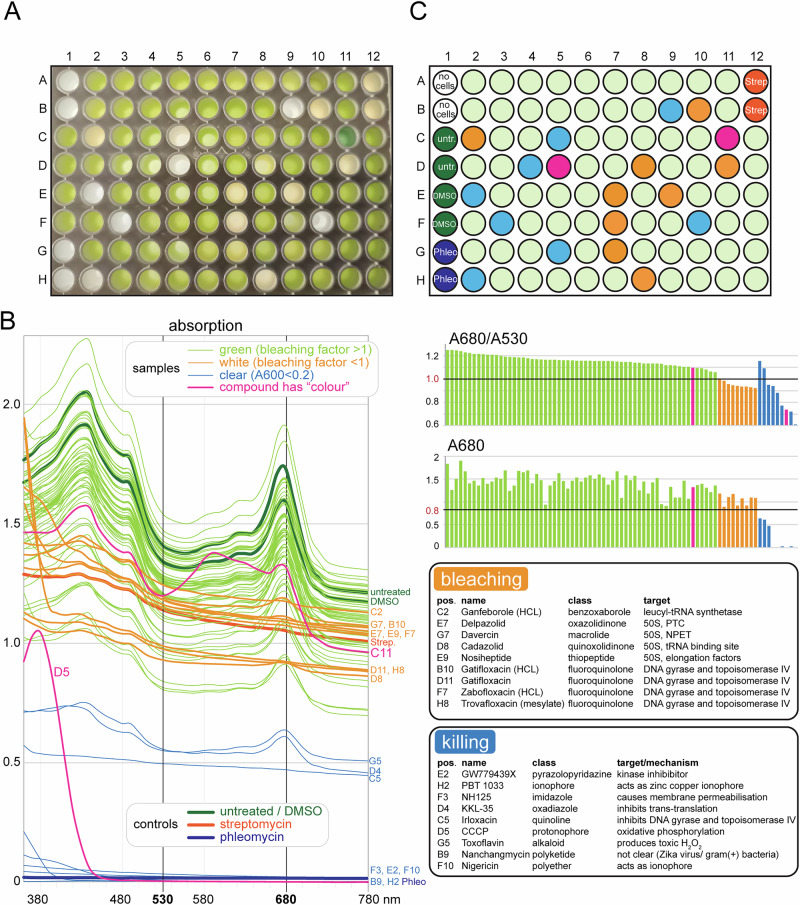
Screen of a commercially available library from MedChemExpress. **A** 96 well plate with *Euglena* treated with compounds (and controls) at the end of the assay (4 days post-plating). **B** Absorption spectra of all wells, coloured according to the outcome of the screen (left). The bleaching value (A680/A530) is blotted for each well and details for bleaching and killing compounds are provided (right). **C** Schematics of the 96-well plate with the wells coloured according to the outcome, using the same colours as in B

Next, we tested the assay on 79 compounds of a commercial library from MedChemExpress (Table S1), which consisted of a mixture of substances with distinct activities to either bacteria or eukaryotes or both. We used the conditions and analysis tools defined above. This resulted in the identification of 9 compounds that caused bleaching and 9 that caused killing (Fig. 2). The remaining 61 compounds had no strong effect on the growth or colour of *Euglena* cultures.

The compounds that caused bleaching were reduced to eight, as one was present in two versions (with and without HCl). Of these eight compounds that caused bleaching, five are known inhibitors of prokaryotic translation. Together with our positive control for bleaching, streptomycin, these compounds belong to six different classes of translation-inhibiting drugs, with distinct targets. Ganfeborole is a benzoxaborole and targets the leucyl-tRNA synthetase of *M. tuberculosis* [33, 34]. The aminoglycoside streptomycin targets mainly the decoding centre of the 30S subunit [35, 36]. The other four compounds target mainly the 50S ribosomal subunit, but at different sites, namely at the peptide-transfer centre, the nascent peptide exit tunnel, the tRNA binding site or by interfering with the elongation factors [37]. The 61 compounds that had no strong effect on *Euglena* contained another eight compounds that mainly target translation: amikacin, spiramycin, linezolid, eperezolid, lexithromycin, sutezolid, lymecycline and isepamicin. These belong to four different chemical classes (aminoglycoside, macrolide, oxazolidinone, tetracycline), of which three overlap with the classes of the compounds that cause bleaching. Thus, a subgroup of compounds that target translation via different mechanisms causes bleaching and this subgroup is not defined by a specific chemical class, consistent with the data of [19]. With ganfeborole, we found the first translational inhibitor with *Euglena*-bleaching capacities that does not act on the ribosome directly.

The remaining three compounds that caused bleaching are all fluoroquinolones and thus affect DNA replication by targeting DNA gyrase and topoisomerase IV [38, 39]. Among the non-bleaching compounds, there is only one other compound that targets DNA replication: Irloxacin. This is a quinolone and its absorption profile (C5 in Fig. 2B) indicates bleaching, but the total absorption is slightly below the threshold chosen to define ‘killing’, indicating that irloxacin is a bleaching compound that is slightly toxic to *Euglena*.

The remaining eight compounds that caused killing did not target translation or DNA replication. Two were ‘mild killers’ at the chosen concentrations: toxoflavin and KKL-35; the remaining compounds were strong killers, allowing no growth of *Euglena*. All compounds either affected eukaryotic-specific processes or fundamental processes of both bacteria and eukaryotes.

Two compounds had absorption profiles distinct to the canonical profiles seen for ‘growing’, ‘bleaching’ or ‘killing’ (shown in pink in Fig. 2). Carbonyl cyanide 3-chlorophenylhydrazone (at plate position D5) had a strong absorption peak at 380 nm, but otherwise no absorption and the automatised classification would have grouped it (correctly) as ‘killing’. Rifalazil (at plate position C11) had a distinct absorption profile in the visible spectrum, but the automatised classification would still have grouped it (correctly) as ‘growing’. Even though, for these two examples, our combination of bleaching-factor and threshold resulted in the correct groupings, coloured substances can potentially result in false positives or false negatives; when the analysis is done exclusively by the bleaching-factor and best to be analysed manually.

### Test on a natural products library

**Fig. 3:**
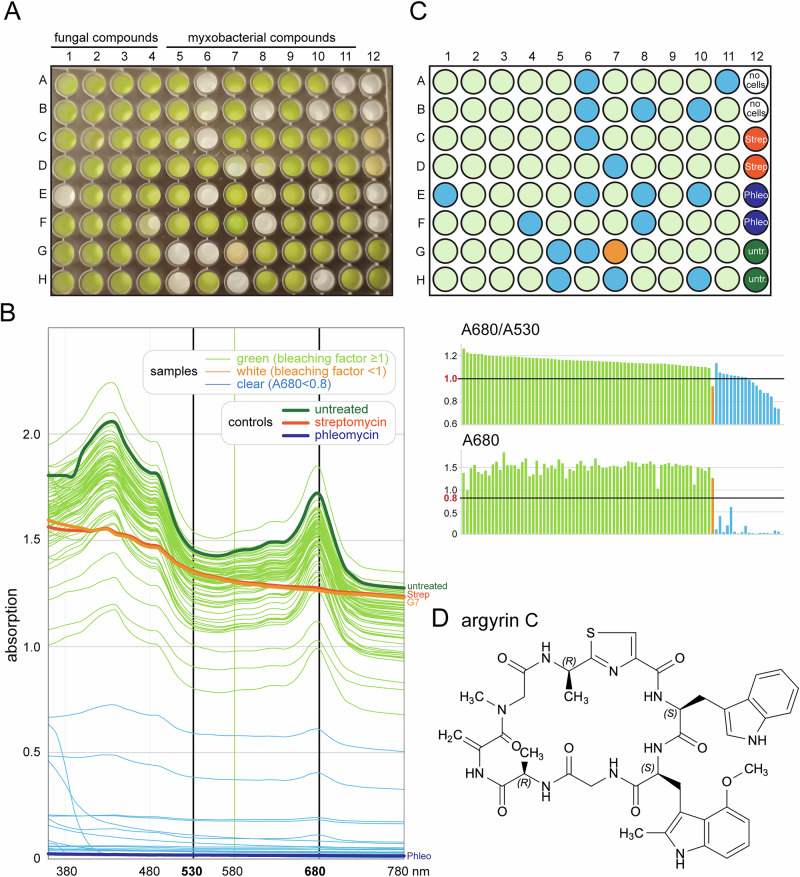
Screen of a library with natural compounds. **A** 96 well plate with *Euglena* treated with the natural compound library (and controls) at the end of the assay (4 days post plating). **B** Absorption spectra of all wells, coloured according to the outcome of the screen (left). The bleaching value (A680/A530) is blotted for each well. **C** Schematics of the 96-well plate with the wells coloured according to the outcome, using the same colours as in (**B**). **D** Structure of argyrin (**C**)

Next, as a proof of principle, we screened a subset of natural products from the compound collection of the Helmholtz Institute for Pharmaceutical Research Saarland (HIPS), using identical conditions (Fig. 3 and Table S2). We selected 88 compounds, of which the majority (56) were myxobacterial metabolites, which constitute a particularly rich source of structurally unique and biologically diverse natural products with high potential for pharmaceutical exploitation. The remaining compounds (32) were of fungal origin and part of the natural products collection of Helmholtz Centre for Infection Research (HZI).

The screening revealed that 18 of the 88 tested natural products prevented the growth of *Euglena* (Fig. 3A–C). Many of these act on well-characterised targets. Some are known to target mitochondria: stigmatellins and aurachins inhibit components of the respiratory chain [40, 41] and cruentarens targets the mitochondrial F₁F₀-ATP synthase [42]. Archazolids and apicularens block vacuolar-type ATPases [43, 44]. Disorazols and tubulysins disrupt microtubule assembly [45, 46] and epothilon A stabilises microtubules [47].

Notably, one compound caused pronounced bleaching of *Euglena* cells. This compound was identified as argyrin C, a myxobacterial cyclic octapeptide belonging to a family of structurally related argyrins first described for their potent antibacterial and antitumor activities [48] (Fig. 3D). Argyrins display a broad spectrum of biological effects, including inhibition of bacterial protein synthesis and modulation of the eukaryotic proteasome pathway, reflecting their high intracellular target specificity. Argyrins are known to inhibit the mitochondrial elongation factor G (FusA1), thereby blocking mitochondrial protein synthesis [49, 50].

To summarise, the *Euglena* screen is applicable to a library of natural products and has identified one compound with a known antibacterial activity.

## Discussion

Novel methods to discover drugs that target pathogenic bacteria are urgently needed, since multi-resistant germs increasingly become a major threat to human health. We here propose to use *Euglena* cells to screen compound libraries for possible antibacterial activity. The novelty and uniqueness of this method is that it can test three important criteria at once: (i) antibacterial effect (bleaching), (ii) no eukaryote and mitochondrial toxicity (*Euglena* lives) and (iii) compound membrane permeability through multiple membranes of different evolutionary origin. Positive hits from this screen therefore have the potential to be effective, non-toxic and able to fight intracellular infections and cross bacterial membranes. To the best of our knowledge, such a combination of outputs cannot be achieved with any other screening method. A further advantage of the method is its cost-effectiveness: *Euglena* grow in water with 0.8% ethanol as a carbon source and traces of salts, vitamin B1 and B12, but do not require serum. They are extremely easy to culture in this simple medium; in fact, they can be kept on a windowsill in a culture flask for at least two months without any attention and they are safety level 0. The screen can be easily upscaled, the hands-on time requirements are short and the total time of the screen is only 4 days. For non-coloured substances, the plates can be read at just two wavelengths to determine both the bleaching factor and growth. For coloured substances, absorption profiles should be recorded and evaluated manually.

The method is applicable to any kind of compound library, with essentially no limitations, except that the compounds shouldn’t be coloured. These can be classical libraries of natural compounds or synthetic compounds or any combination of both platforms, aided by modern chemistry and neural-network-based predictions. It is not limited to a specific target and thus has the potential to discover drugs with novel targets. On the downside, the *Euglena*-based screen will not identify compounds that target pathways not preserved in the chloroplast (false negatives). Obvious false negatives are drugs that target the synthesis of the cell wall, since chloroplasts lack a cell wall. However, the *Euglena* chloroplast has independently evolved from bacterial pathogens over a long time and will thus also contain differences to bacteria in pathways that are present, such as translation or transcription. Of 13 drugs that primarily target translation, 5 were bleaching *Euglena* and 8 were not, indicating ~60% false negatives in this particular pathway. In contrast, DNA gyrase and topoisomerase IV appear more conserved, as 3 out of 4 quinolones caused bleaching and the remaining one, in principle, as well, just with a slight toxic effect.

Moreover, there is the possibility that some compounds specifically target photosynthesis rather than essential bacteria-derived processes (false positives). Therefore, the *Euglena* screen is located at the beginning of a development pipeline for drugs against bacterial infections. It is optimised for high-throughput screenings and the outcome is a list of compounds for further downstream tests.

In particular, Gram-negative bacteria remain a challenge to treat and increasingly develop resistance. Of the three pathogen groups that the WHO classifies as the most critical ones to become untreatable in the near future due to resistance, two are Gram-negative strains (Enterobacterales and *Acinetobacter baumannii*) [51]. The second high-priority group contains seven bacterial strains, of which five are Gram-negative (*Salmonella Typhi, Shigella spp*., *Pseudomonas aeruginosa*, *Non-typhoidal Salmonella* and *Neisseria gonorrhoeae*) [51]. The *Euglena* chloroplast originates from the Gram-negative cyanobacteria, like all chloroplasts. Consistent, it is targeted by antibiotics known to be effective against Gram-negative strains, like streptomycin and ofloxacin. Of the eight antibiotics that we found to bleach *Euglena*, four are effective against Gram-negative bacteria (ganfeborole and the three fluoroquinolones). Thus, *Euglena* provides a suitable platform to screen for compounds targeting Gram-negative bacteria.

Compounds that target either the ribosome or the DNA gyrase and topoisomerase IV had been previously known to bleach *Euglena* [19]. In this work, we have identified two new compounds that bleach *Euglena*. One is the benzoxaborole ganfeborole which targets the leucyl-tRNA synthetase of *M. tuberculosis* [33, 34] and is in phase 2a clinical trials for the treatment of patients with rifampicin-resistant strains [52]. The second is argyrin C. Related compounds have a broad spectrum of biological effects. Argyrins target the mitochondrial elongation factor G [49, 50]. The discovery of two novel compounds with bleaching activity in *Euglena* proves that the screen has the potential to identify antibacterial drugs with targets beyond ribosomes and transcription.

Multi-resistant pathogens are likely to become the leading cause of death in the next decades and new methods to screen for drugs that target these pathogens are urgently needed. With *Euglena*, we here add a new tool for large, initial screens that combines toxicity, membrane permeability and antibacterial activity in a single assay.

## Supplementary information

Figure S1

Table S1

Table S2
