# Supplementary figures and images for: Euglena gracilis as a high-throughput screening platform for antibacterial activity, cytotoxicity and membrane permeability in a one-step and cost-effective assay
Source: J Antibiot (Tokyo). 2026 Mar 18;79(6):376–85. doi: 10.1038/s41429-026-00911-5 (PMC13212157; doi:10.1038/s41429-026-00911-5)

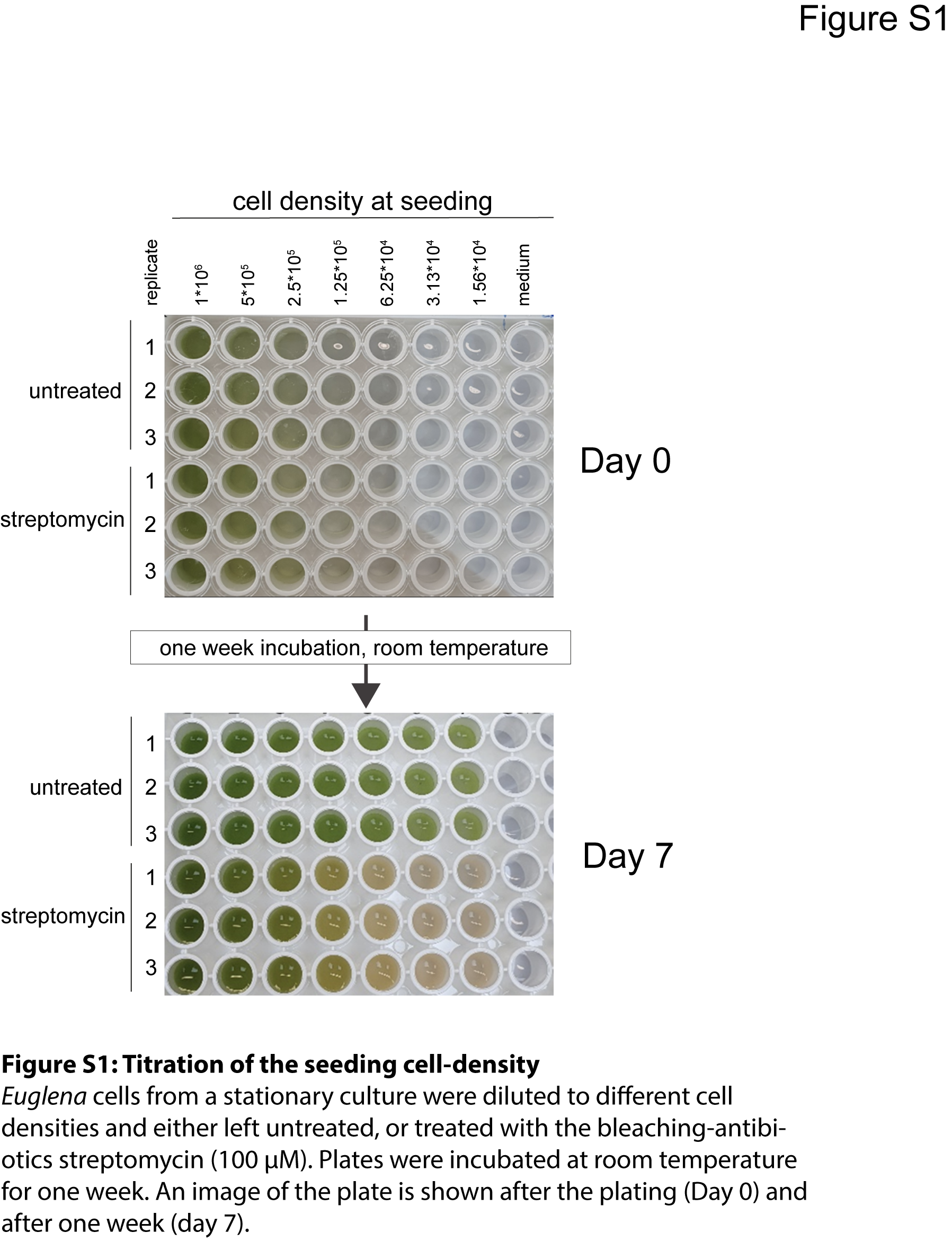

Supplement: Supplementary file 1 — Figure S1 [file 41429_2026_911_MOESM1_ESM.tif]
