# Supplementary material for: Euglena gracilis as a high-throughput screening platform for antibacterial activity, cytotoxicity and membrane permeability in a one-step and cost-effective assay
Source: J Antibiot (Tokyo). 2026 Mar 18;79(6):376–85. doi: 10.1038/s41429-026-00911-5 (PMC13212157; doi:10.1038/s41429-026-00911-5)
Supplement: Supplementary file 3 — Table S2 [file 41429_2026_911_MOESM3_ESM.docx]

**Table S2: Fungal and myxobacterial natural products from the HZI/HIPS libraries used for bioactivity screening.**

| **Compound name** | **Molecular weight [Da]** | **Origin** | **SMILES** |
| --- | --- | --- | --- |
| **10,11-Dehydrocurvularin** | 290.3111 | fungal | O=C1Cc2cc(O)cc(O)c2C(=O)C=CCCC[C@H](C)O1 |
| **5-Methylcarbonylmellein** | 236.223 | fungal | O=C(OC)c1ccc(O)c2C(=O)O[C@@H](C)Cc12.O=C(OC)c1ccc(O)c2C(=O)O[C@@H](C)Cc12 |
| **8-O-Acetoxymultiplolide A** | 256.2518 | fungal | CC(=O)O[C@@H]1C[C@@H](C)OC(=O)[C@H]2O[C@H]2C=C[C@@H]1O |
| **Aureochinone** | 250.2042 | fungal | Oc1c(O)c(C)c(O)c2C(=O)C(C)=C(O)C(=O)c12 |
| **Compound 248** | 248.3175 | fungal |  |
| **Connatusin B** | 266.3328 | fungal | O=C1C(O)=C(C)[C@@]2(C)[C@H]3[C@@H](C[C@@]12O)C[C@@](C)(C)[C@@H]3O |
| **Curvulin** | 238.2366 | fungal | Oc1cc(O)cc(CC(=O)OCC)c1OC(C)=O |
| **Curvulinic acid** | 210.1834 | fungal | O=C(C)c1c(cc(O)cc1O)CC(=O)O |
| **Daldinone A** | 336.338 | fungal | Oc1cccc2c1C(=O)C[C@H]1[C@H]3CCC(=O)c4c(O)ccc(c43)[C@]12O |
| **Deoxyphomalone** | 238.2796 | fungal | CCc1c(O)cc(OC)c(C(=O)CCC)c1O |
| **Deschlorozopfinol** | 306.3966 | fungal | OCc1c(cccc1O)/C=C/C(O)C(O)\C=C\CCCCC |
| **Eburicoic acid** | 470.7269 | fungal | O=C(O)[C@@H](CCC(=C)C(C)C)[C@H]1CC[C@@]2(C)C=3CC[C@@H]4[C@](C)(CC[C@H](O)[C@@]4(C)C)C=3CC[C@]12C |
| **Hypoxylan A** | 220.3074 | fungal | CC(CO)c1cc2c(CCC[C@@H]2C)cc1O |
| **Ionylideneacetic acid** | 234.334 | fungal | CC1(C)CCCC(=C)[C@H]1/C=CC(\C)=C\C(=O)O |
| **Massarilactone H** | 224.21 | fungal | C=C1OC(=O)C2=C1OC(\C=C\C)C(O)C2O |
| **Myrocin B** | 358.3851 | fungal | C[C@]12CC[C@H]3C[C@@]33[C@H]1[C@](O)(OC2=O)C(=O)C1=C[C@](C)(CC(=O)[C@@]13O)C=C |
| **Orsellinic acid** | 168.1467 | fungal | OC(=O)c1c(C)cc(O)cc1O |
| **Pereniporin A** | 268.3487 | fungal | CC1(C)CCC[C@@]2(C)[C@H]1[C@H](O)C=C1CO[C@@H](O)[C@@]12O |
| **Phenylethylanthranilate** | 241.2851 | fungal | Nc1ccccc1C(=O)OCCc1ccccc1 |
| **Phlebopyrone** | 296.2742 | fungal | O=C1OC=C(C(O)=C1c1ccc(O)cc1)c1ccc(O)cc1 |
| **Rhodocorane I** | 236.3068 | fungal | CC1=C/C(OC1=O)=C1\[C@H](CC[C@H]1C)[C@H](C)CO |
| **Rhodocorane J** | 236.3068 | fungal | CC1=C\C(OC1=O)=C1\[C@H](CC[C@H]1C)[C@@H](C)CO |
| **Rickenyl C** | 382.406 | fungal | Oc1c(c(O)c(OC)c(c2ccc(OC)cc2)c1OC)c1ccc(OC)cc1 |
| **Rickiol A** | 428.6026 | fungal | C[C@@H](O)C[C@@H](O)C[C@@H]1CCCCCCCCCCCC[C@H](O)C[C@H](O)C=CC(=O)O1 |
| **Rosellisin** | 270.2354 | fungal | OCC1=C(OC)C(CO)=C(/C=C/C(=O)OC)OC1=O |
| **Sporothriolide** | 238.2796 | fungal | O=C1O[C@H](CCCCCC)[C@@H]2OC(=O)C(=C)[C@H]12 |
| **TMC 256A1** | 272.2528 | fungal | OC=1C=C(C)OC2=CC3=CC(=O)C=C(OC)C3=C(O)C2=1 |
| **Trichodion** | 238.2366 | fungal | C[C@@H]1OC=2O[C@H](\C=C\C)[C@@H](O)C(=O)C=2C(=O)C1 |
| **Trichothecolone acetate** | 306.3536 | fungal | CC(=O)O[C@@H]1CC2OC3C=C(C)C(=O)C[C@]3(C)[C@]1(C)C12CO1 |
| **Truncatone variant** | 332.3063 | fungal |  |
| **Viriditin A** | 307.4278 | fungal | OC[C@@H]1CCCN1C(=O)\C=C\C=C\C(\C)=C\[C@H](O)CCCC |
| **Vulculic acid** | 240.2094 | fungal | COc1cc(CC(=O)O)c(c(O)c1O)C(C)=O |
| **Acetophenone variant 219** | 219.2796 | myxobacterial |  |
| **Acetophenone variant 235** | 235.279 | myxobacterial |  |
| **Acetophenone variant 249** | 249.3056 | myxobacterial |  |
| **Ajudazol A** | 590.7065 | myxobacterial | C\C(OC)=C/C(=O)N(C)C\C=C\CC\C=C/C=C\CC(=C)c1occ(n1)C[C@H](C)[C@@H]1OC(=O)c2c(ccc(C)c2O)[C@H]1O |
| **Ambruticin A** | 472.6136 | myxobacterial | CC1=CC[C@@H](O[C@@H]1CC)C(/C)=C/[C@H](C)/C=C/[C@@H]1[C@@H](/C=C/[C@@H]2O[C@@H](CC(=O)[C@H]2O)CC(=O)O)[C@@H]1C |
| **Ambruticin F** | 474.6295 | myxobacterial | CC1=CC[C@@H](O[C@@H]1CC)C(/C)=C/[C@H](C)/C=C/[C@@H]1[C@@H](/C=C/[C@@H]2O[C@@H](C[C@@H](O)[C@H]2O)CC(=O)O)[C@@H]1C |
| **Ambruticin VS-3 N-Oxid** | 517.6973 | myxobacterial | CC1=CC[C@@H](O[C@@H]1CC)C(/C)=C/[C@H](C)/C=C/[C@@H]1[C@@H](/C=C/[C@@H]2O[C@@H](C[C@@H]([C@H]2O)[N+]([O-])(C)C)CC(=O)O)[C@@H]1C |
| **Aminopyrrolnitrin** | 227.0899 | myxobacterial | Clc1c[NH]cc1c1cccc(Cl)c1N |
| **Angiolam B** | 585.7712 | myxobacterial | C=C\C=C(/C)C(=O)CC[C@H](C)/C=C/[C@H]1CC[C@H](C)C(=O)CC(=O)N[C@H](C)CCC(=O)[C@H](C)[C@@H](O)[C@H](C)C=C(C)C(=O)O1 |
| **Antalid** | 527.6755 | myxobacterial | CC(C)[C@@H]1NC(=O)C(C)=C[C@@H](C)[C@H](OC(=O)[C@H](NC(=O)c2nc1sc2)Cc1ccccc1)C[C@@H](O)CC |
| **Apicularen A** | 441.5168 | myxobacterial | CC\C=C/C=C\C(=O)N\C=C\C[C@@H]1OC(=O)c2c(O)cccc2C[C@H]2C[C@@H](O)C[C@H](C1)O2 |
| **Archazolid B** | 724.9893 | myxobacterial | O=C(OC(CC(C)C)c1nc(cs1)C1OC(=O)C=CCC(C)=CC(O)C(C)C=C(C)C=C(C)C=CC(O)C(C)C(OC)C(C)=CC=CC1C)NC |
| **Argyrin C** | 838.9312 | myxobacterial | COc1cccc2[NH]c(C)c(C[C@@H]3NC(=O)[C@@H](NC(=O)c4nc(sc4)[C@@H](C)NC(=O)CN(C)C(=O)C(=C)NC(=O)[C@@H](C)NC(=O)CNC3=O)Cc3c[NH]c4ccccc43)c12 |
| **Aurachin A** | 395.5344 | myxobacterial | C/C(C)=C\CCC(\C)=C\CCC(C)(O)C1Cc2c(O1)c(C)[n+]([O-])c1ccccc12 |
| **Aurafuron A** | 378.5024 | myxobacterial | CC1=C(CC(O)/C=C\C=C\C(C)C(O)C(\C)=C\CC(C)C)OC(C)(O)C1=O |
| **Bengamide variant** | 372.4565 | myxobacterial |  |
| **Carolacton** | 468.5803 | myxobacterial | O=C(O)CC(OC)C(C)C(=O)C(C)\C=C(/C)C1OC(=O)C(O)C(O)C=CC(C)CCCC1C |
| **Chivosazol A** | 866.0876 | myxobacterial | CC(O)CC(O)C(C)C1OC(=O)C=CC=CC=CC(C)=CC(C)C(OC2O[C@H](C)[C@@H](O)[C@H](OC)[C@H]2OC)C=CC=Cc2nc(oc2)C(C)C(OC)CC(O)C=CC=CC=CC1C |
| **Chondrochloren A** | 526.062 | myxobacterial | Oc1ccc(/C=C\NC(=O)[C@H](OC)[C@H](O)[C@@H](/C=C(\C)C(=O)[C@@H](C)[C@@H](O)[C@H](C)CCCC)OC)cc1Cl |
| **Chondrodepsid A** | 831.9536 | myxobacterial | Oc1ccc(cc1)C[C@@H](NC(=O)\C=C\CCc1ccccc1)C(=O)N[C@@H]1C(=O)N[C@@H](C)C(=O)N2CCC[C@H]2C(=O)N[C@H]([C@@H](C)CC)C(=O)N[C@@H](CCC(N)=O)C(=O)O[C@@H]1C |
| **Cittilin A** | 630.6875 | myxobacterial | O=C(O)C1NC(=O)C2Cc3cc(c4cc(ccc4OC)CC(N)C(=O)NC(C(C)CC)C(=O)N2)c(O)c(c3)Oc2ccc(C1)cc2 |
| **Compound 322** | 322.4424 | myxobacterial |  |
| **Compound 464** | 464.638 | myxobacterial |  |
| **Compound 487** | 487.6282 | myxobacterial |  |
| **Crocadepsin A** | 872.3171 | myxobacterial | NC(=O)[C@H](O)[C@@H]1NC(=O)[C@@H](C)[C@H](O)[C@@H](NC(=O)C(=C\C)/NC(=O)/C=C/c2ccccc2)[C@H](OC(=O)[C@H](CC(=O)O)NC(=O)[C@@H](N)[C@@H](C)CCNC1=O)c1ccc(OC)c(Cl)c1 |
| **Cruentaren A** | 589.7599 | myxobacterial | CCCC(O)C(C)C(=O)NC\C=C\CC(C)C(O)C(C)C1CC=CCC(C)C(O)Cc2cc(OC)cc(O)c2C(=O)O1 |
| **Disorazole A** | 758.8963 | myxobacterial | C\C=C\C(O)C(C)(C)C1CC=CC=CC=CC(OC)Cc2occ(n2)C(=O)OC(CC=CC2OC2C=CC=Cc2occ(n2)C(=O)O1)C(C)(C)C(O)\C=C\C |
| **Disorazole A2** | 744.8697 | myxobacterial | C\C=C\C(O)C(C)(C)C1CC=CC=CC=CC(O)Cc2occ(n2)C(=O)OC(CC=CC2OC2C=CC=Cc2occ(n2)C(=O)O1)C(C)(C)C(O)\C=C\C |
| **Disorazole B1** | 742.8538 | myxobacterial | C\C=C\C(O)C(C)(C)C1CC=CC2OC2C=CC=Cc2occ(n2)C(=O)OC(CC=CC2OC2C=CC=Cc2occ(n2)C(=O)O1)C(C)(C)C(O)\C=C\C |
| **Elansolid A2** | 588.7734 | myxobacterial | Oc1ccc(cc1)C1OC(=O)C=CC(C)=CCC(O)C(C)C(O)C=CC=CC=CC2C1C1C(C=C2C)[C@@](C)(O)C[C@@]1(C)C |
| **Elansolid C1** | 725.9094 | myxobacterial | O=C(O)/C=CC(\C)=C\CC(O)C(C)C(O)/C=C\C=C\C=C/C1C(C2C(C=C1C)[C@@](C)(O)C[C@@]2(C)C)C(Nc1ccccc1C(=O)O)c1ccc(O)cc1 |
| **Epothilone A** | 493.656 | myxobacterial | Cc1nc(/C=C(\C)[C@@H]2C[C@@H]3O[C@@H]3CCC[C@H](C)[C@H](O)[C@@H](C)C(=O)[C@](C)(C)[C@@H](O)CC(=O)O2)cs1 |
| **Gephyronic acid A** | 470.6393 | myxobacterial | OC(C(C)CC(C)C(=O)C(C)(C)C(OC)C(C)C(O)CC(=O)O)C1(C)OC1C(C)\C=C(/C)C |
| **Glidobactin A** | 520.6614 | myxobacterial | C[C@@H](O)[C@H](NC(=O)\C=C\C=C\CCCCCCC)C(=O)N[C@H]1CC(O)CCNC(=O)C=C[C@H](C)NC1=O |
| **Haprolid** | 682.8897 | myxobacterial | CC(C)C[C@@H]1C(=O)N2CCC[C@H]2C(=O)O[C@@H](CCC(C)=CCC(=O)N[C@@H](Cc2ccccc2)C(=O)N(C)CC(=O)N1C)CCC[C@@H](C)OC |
| **Hyaladione** | 203.646 | myxobacterial | NC=1C(=O)C=C(SC)C(=O)C=1Cl |
| **Imidacin A1** | 318.4537 | myxobacterial | O=C(O)C1CC1CCCCCCCCCC\C=C\c1c[NH]cn1 |
| **Jerangolid E** | 362.503 | myxobacterial | C[C@H]1CC[C@@H](O[C@@H]1CC)C(\C)=C\[C@H](C)/C=C/[C@H]1CC(OC)=C(C)C(=O)O1 |
| **Jerangolid H** | 378.5024 | myxobacterial | C[C@H]1CC[C@@H](O[C@@H]1CC)C(\C)=C\[C@H](C)/C=C/[C@H]1CC(OC)=C(CO)C(=O)O1 |
| **Leupyrrin A1** | 738.9066 | myxobacterial | CC(C)CC1CC(=O)OC2C(OC(=O)C2(C)CO)C(C)=CCc2ccc([NH]2)C=2OC(COC1=O)C(N=2)/C=C/1\C(CC(C)C)OC/C\1=C(/C)CCCOC |
| **Lipid-Symbioramid** | 581.9532 | myxobacterial | O=C(N[C@@H](CO)[C@H](O)CCCCCCCCCCCCCCC)[C@H](O)\C=C\CCCCCCCCCCCCCC |
| **Myxalamid S** | 419.5974 | myxobacterial | C\C(=C/[C@@H](C)[C@@H](O)C(/C)=C/C(C)C)\C=C\C(O)C\C=C\C=C(/C)C(=O)N[C@@H](C)CO |
| **Myxochelin A** | 404.4138 | myxobacterial | Oc1c(cccc1O)C(=O)NC(CCCCNC(=O)c1cccc(O)c1O)CO |
| **Myxothiazol A** | 487.6778 | myxobacterial | C[C@@H](\C=C\C=C\C(C)C)c1nc(cs1)c1scc(/C=C/[C@H](OC)[C@@H](C)C(=C\C(N)=O)/OC)n1 |
| **Myxovirescin A** | 623.8608 | myxobacterial | O=C1O[C@@H](CCC)C(=O)NC[C@@H](O)C[C@H](O)[C@H](O)CCC(COC)=CC=C[C@H](CC)CCCC(=O)CCCC[C@@H](C)C[C@@H]1C |
| **Noricumazol B** | 633.7264 | myxobacterial | CC(CC)Cc1ccc2CC(OC(=O)c2c1O)CC(O)/C=C/c1nc(oc1)CC(OC1OC(CO)C(O)C1O)C(C)C(O)CC |
| **Pellasoren A** | 431.6081 | myxobacterial | CC\C=C(/OC)C(=O)N[C@@H](C)\C=C(/C)\C=C(/C)\C=C\C[C@H](C)[C@H]1OC(=O)[C@H](C)C[C@@H]1C |
| **Ratjadon A** | 456.6142 | myxobacterial | OC1CC(OC(\C=C\C)C1C)C(O)\C=C\C=C(/C)CC(C)/C=C(/C)\C=C\C1CC=CC(=O)O1 |
| **Ripostatin A** | 494.6191 | myxobacterial | O=C(O)CC=1CC=CCC=C(C)CC2C[C@@H](OC(=O)C=1)C[C@@](O)(CCC(\C)=C\Cc1ccccc1)O2 |
| **Saframycin Mx1 variant** | 566.6023 | myxobacterial |  |
| **Stigmatellin A** | 514.6503 | myxobacterial | C\C=C(/C)\C=C\C=C\[C@@H](OC)[C@@H](C)[C@@H](OC)[C@@H](C)CCC=1Oc2c(O)c(cc(OC)c2C(=O)C=1C)OC |
| **Terrestribisamid A** | 440.4889 | myxobacterial | Oc1ccc(cc1OC)/C=C/C(=O)NCCCCNC(=O)/C=C/c1ccc(O)c(OC)c1 |
| **Tubulysin A** | 844.0687 | myxobacterial | CN1CCCC[C@@H]1C(=O)N[C@H](C(=O)N(COC(=O)CC(C)C)[C@H](C[C@@H](OC(C)=O)c1scc(n1)C(=O)N[C@@H](Cc1ccc(O)cc1)C[C@H](C)C(=O)O)C(C)C)[C@@H](C)CC |
| **Tubulysin A variant** | 860.0681 | myxobacterial |  |
| **Vioprolide A** | 863.0323 | myxobacterial | CC(C)C[C@H]1NC(=O)[C@H](C)NC(=O)[C@@H](O)COC(=O)[C@H](C(C)C)N(C)C(=O)[C@@H](NC(=O)[C@@H]2CCCCN2C(=O)C(=C\C)/NC(=O)[C@H]2N=C(SC2)[C@@H]2C[C@@H](C)N2C1=O)C(C)O |
| **Vioprolide D** | 849.0057 | myxobacterial | CC(C)C[C@H]1NC(=O)[C@H](C)NC(=O)[C@@H](O)COC(=O)[C@H](C(C)C)N(C)C(=O)[C@@H](NC(=O)[C@@H]2CCCN2C(=O)C(=C\C)/NC(=O)[C@H]2N=C(SC2)[C@@H]2CCCN2C1=O)C(C)O |

All compounds were isolated from fungal or myxobacterial origin. prepared as DMSO stock solution with a concentration of 10 mM each.

NUR ZUR INFO, GEHÖRT NICHT ZUR TABELLE

Eighty-eight isolated natural products from the HZI/HIPS natural products libraries (32 fungal and 56 myxobacterial compounds) were used for bioactivity screening in a novel *Euglena* model. Molecular weight (MW) and SMILES notation are given where available. Stock solutions were prepared at 10 mM in DMSO. For compounds whose chemical structures have not yet been elucidated, the corresponding fields are left empty.
